# Changes in Serum Growth Factors during Lenvatinib Predict the Post Progressive Survival in Patients with Unresectable Hepatocellular Carcinoma

**DOI:** 10.3390/cancers14010232

**Published:** 2022-01-04

**Authors:** Zijian Yang, Goki Suda, Osamu Maehara, Masatsugu Ohara, Sonoe Yoshida, Shunichi Hosoda, Megumi Kimura, Akinori Kubo, Yoshimasa Tokuchi, Qingjie Fu, Ren Yamada, Takashi Kitagataya, Kazuharu Suzuki, Naoki Kawagishi, Masato Nakai, Takuya Sho, Mitsuteru Natsuizaka, Kenichi Morikawa, Koji Ogawa, Shunsuke Ohnishi, Naoya Sakamoto

**Affiliations:** 1 Department of Gastroenterology and Hepatology, Graduate School of Medicine, Hokkaido University, Sapporo 060-8589, Japan; yuang@eis.hokudai.ac.jp (Z.Y.); masamasama_zu@yahoo.co.jp (M.O.); sonoeds@pop.med.hokudai.ac.jp (S.Y.); hosoda.shunichi.k0@elms.hokudai.ac.jp (S.H.); myamakam@med.hokudai.ac.jp (M.K.); kubo.akinori.q5@elms.hokudai.ac.jp (A.K.); h.y.tokuchi112@med.hokudai.ac.jp (Y.T.); fuqingjie@pop.med.hokudai.ac.jp (Q.F.); renyama5@pop.med.hokudai.ac.jp (R.Y.); t.kitagataya@pop.med.hokudai.ac.jp (T.K.); kazuharu-s@hospital.hakodate.hokkaido.jp (K.S.); naopaleg@yahoo.co.jp (N.K.); mnakai@pop.med.hokudai.ac.jp (M.N.); shotaku@pop.med.hokudai.ac.jp (T.S.); mitsuteru1975@huhp.hokudai.ac.jp (M.N.); kenichi.morikawa@med.hokudai.ac.jp (K.M.); k-ogawa@med.hokudai.ac.jp (K.O.); 2 Laboratory of Molecular and Cellular Medicine, Faculty of Pharmaceutical Sciences, Hokkaido University, Sapporo 060-0812, Japan; maeosa17@frontier.hokudai.ac.jp (O.M.); sonishi@pop.med.hokudai.ac.jp (S.O.)

**Keywords:** post progressive survival, lenvatinib, HCC, growth factors, prognosis

## Abstract

**Simple Summary:**

In this study, we firstly revealed that the pattern of developing resistance to lenvatinib varies and determines the prognosis of patients with unresectable hepatocellular carcinoma (HCC) by analyzing the changes in growth factors during lenvatinib for unresctable HCC. The evaluation of changes in growth factors during lenvatinib could predict treatment response and PPS and could be used for the determination of salvage therapy.

**Abstract:**

Serum growth factor changes and their effect on prognosis during lenvatinib for unresectable hepatocellular carcinoma (HCC) remain underexplored. The sequential changes in serum growth factors during lenvatinib for unresectable HCC were evaluated in 58 patients using complete clinical data, and preserved serum was used to investigate changes in FGF-19, ANG-2, HGF, VEGF, and EGF. Patients with a complete response (CR), partial response (PR), and stable disease (SD) were evaluated for growth factor changes between the best response and progressive disease (PD) points, classified based on these changes, and evaluated by post progression survival (PPS). A total of 8, 24, 18, and 8 patients showed CR, PR, SD, and PD, respectively. Multivariate analysis revealed that age, relative dose intensity, and baseline ANG-2 were significantly associated with treatment response. Growth factor changes between the best response and PD points revealed that patients could be classified into four groups based on the EGF, ANG-2, and HGF changes. Although patient characteristics at baseline and PD, their response to lenvatinib, and PFS were similar among those groups, patients with an increase in all growth factors had significantly shorter PPS (median PPS was 553, 323, and 316 versus 173 days in groups 1–4 *p* = 0.032). We revealed that the evaluation of the changes in growth factors during lenvatinib could predict PPS.

## 1. Introduction

Hepatocellular carcinoma (HCC) is the fourth leading cause of cancer-related deaths, and its incidence has been increasing globally [1]. Until recently, therapeutic options for unresectable HCC were limited to the multi-tyrosine kinase inhibitors (TKIs) of sorafenib [2]. However, various therapeutic options for unresectable HCC have lately become available due to successful clinical trials with novel drugs, including TKIs of sorafenib, lenvatinib, regorafenib, cabozantinib, monoclonal antibodies against vascular endothelial growth factor receptor (VEGFR)-2 of ramucirumab, and immune checkpoint inhibitors (ICIs) [3,4,5,6,7].

Lenvatinib is a potent TKI for unresectable HCC and mainly targets fibroblast growth factor receptors (FGFR)-1–4, VEGFR-1–3, and c-Kit, and rearranges during transfection proto-oncogene [8,9]. Phase three of the clinical REFLECT trial and real-world data have shown the efficacy and safety of lenvatinib for unresectable HCC [6,10,11,12,13,14,15]. In the REFLECT trial, the lenvatinib treatment group had higher overall survival (OS) and median progression-free survival (PFS) than the sorafenib treatment group. The American Society of Clinical Oncology guidelines state that lenvatinib and sorafenib should be the first-line therapy in patients with unresectable HCC contraindicated to atezolizumab and bevacizumab and second-line therapy in patients who fail to respond to atezolizumab and bevacizumab [16].

Although lenvatinib can suppress FGFR-1–4 and the VEGF signaling pathway, it cannot inhibit hepatocyte growth factor (HGF)-, epidermal growth factor (EGF)-, and angiopoietin-2 (ANG-2)/Tie-2-mediated signaling pathways. Recent reports revealed that high baseline ANG-2 levels are associated with poor response to lenvatinib in unresectable HCC [11] and shorter PFS in advanced medullary thyroid cancer [17]. In addition, high baseline HGF levels are associated with shorter PFS in lenvatinib therapy for advanced medullary thyroid cancer [17]. In vitro analysis revealed that HGF suppresses the anti-tumor effect of lenvatinib on HCC cell lines with high expression levels of c-MET [18]. Recent reports have revealed that the activation of EGF receptor (EGFR)-mediated signaling reduces the anti-tumor effect of lenvatinib in HCC [19]. Thus, the activation of these signaling pathways, which lenvatinib cannot suppress, might cause resistance to lenvatinib.

To date, the data of sequential changes in growth factors during lenvatinib treatment, especially with respect to progressive disease (PD), have not been completely clarified. These data can provide insights into the acquisition of lenvatinib resistance in unresectable HCC and present important information for the decision of salvage treatment after lenvatinib administration. Therefore, in the present study, we aimed to investigate the sequential changes in serum growth factors during lenvatinib treatment for unresectable HCC.

## 2. Materials and Methods

### 2.1. Patients and Study Design

In the present study, we screened patients who were treated with lenvatinib for unresectable HCC between April 2018 and May 2021 at Hokkaido University Hospital, Sapporo, Hokkaido, Japan. Patients were included if they had complete clinical data, preserved serum for analysis of growth factors at baseline, best response and progressive disease point, were followed for >2 months after the start of lenvatinib treatment, and had an evaluated treatment response using dynamic computed tomography (CT) or dynamic magnetic resonance imaging (MRI) at baseline and every 2–3 months. We excluded patients who were treated with a concomitant use of lenvatinib and other anti-HCC agents, followed for less than 2 months, were not sufficiently evaluated for treatment response, had insufficient clinical information, or had insufficient preserved serum samples for analysis of growth factors.

We collected clinical information including age, sex, laboratory data, tumor markers of α-fetoprotein (AFP) and des-gamma-carboxy prothrombin, liver functional reserve (Child–Pugh score, modified albumin–bilirubin grade), Barcelona Clinic Liver Cancer (BCLC) stage, and serum levels of FGF-19, ANG-2, VEGF, HGF, and EGF at baseline and during and after lenvatinib treatment. Treatment response was evaluated every 2–3 months using dynamic CT or MRI according to the modified response evaluation criteria in solid tumors [20]. PFS was defined as the duration from the day of lenvatinib initiation to the day when disease progression or death was observed [14]. PPS was defined as the duration of disease progression from lenvatinib treatment to death [21]. Objective response was defined as the rate of the patients with complete response or partial response evaluated by mRECIST [22]. Best response was defined as the best response point across all time points up to progression.

Relative dose intensity (RDI) during the initial 8 weeks of therapy was defined as follows: the cumulative dose within the 8 weeks of lenvatinib treatment divided by the standard dose. This study conformed to the ethical guidelines of the Declaration of Helsinki and the study protocol was approved by the ethics committee of Hokkaido University Hospital (approval number: 017-0521). All recruited patients provided written informed consent for participation in the clinical study.

### 2.2. Analysis of Changes in Serum Growth Factors

Serum VEGF, ANG-2, FGF-19, HGF, and EGF levels were evaluated using commercial enzyme-linked immunosorbent assays (R&D Systems, Minneapolis, MN, USA) according to the manufacturer’s protocols [11].

Changes in serum growth factors were analyzed at baseline, best response, and PD points. The rate of growth factor changes between the best response and progressive points in patients with stable disease (SD), partial response (PR), and complete response (CR) were visualized using Heatmapper and classified using average linkage clustering methods [23]. Classified groups were analyzed for the patient characteristics at baseline and at PD point and for PFS and post progression survival (PPS).

### 2.3. Treatment Protocol

During lenvatinib treatment, patients were administered 8 or 12 mg of lenvatinib once a day according to body weight (8 mg for patients with body weight < 60 kg and 12 mg for patients with body weight ≥ 60 kg). Treatment was discontinued when disease progression was observed or unacceptable adverse events occurred. In addition, the attending physician adjusted the dosage of the anti-tumor drugs according to tolerability or adverse events.

### 2.4. Statistical Analysis

In the present study, categorical variables were analyzed using the chi-squared and Fisher’s exact tests, and continuous variables were analyzed using the paired *t*-test, Mann–Whitney *U* test and Wilcoxon signed-rank test. Differences among three or more populations were compared using one-way analysis of variance followed by Tukey’s test. The rate of growth factor changes between the best response and PD points in patients with SD, PR, and CR were visualized using a heat map and clustered by average linkage clustering methods using Heatmapper [23]. Survival curves of PFS and PPS were calculated using Kaplan–Meier analysis and compared using the log-rank test. Multivariate logistic regression analysis was performed with variables regarded as significant at *p* < 0.05 in the univariate analyses. Univariate Cox regression analysis was conducted for the clinical and growth factors and laboratory data; multivariate Cox regression analysis was conducted for the factors showing significance (defined at *p* < 0.05) in the univariate analysis. Statistical analyses were performed using GraphPad Prism version 8.02 (GraphPad Software, La Jolla, CA, USA). For all statistical analyses, *p* < 0.05 was considered statistically significant.

## 3. Results

### 3.1. Patient Characteristics

**Figure 1:**
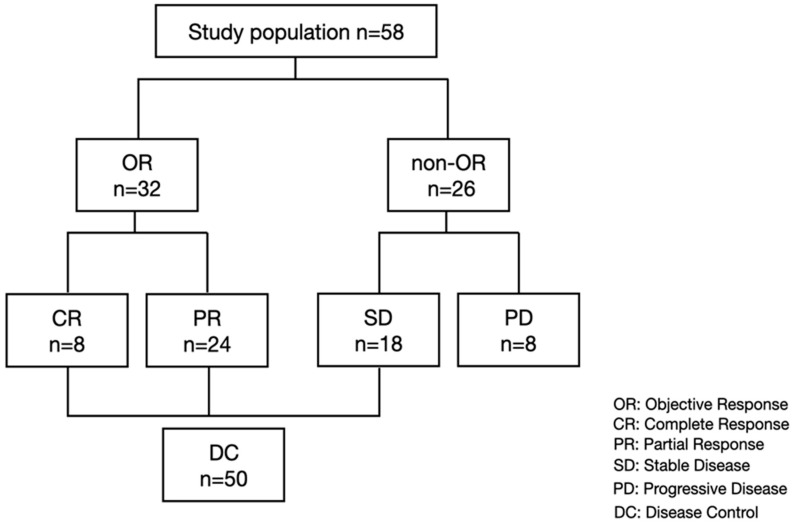
Study flow and treatment response.

In the present study, we included a total of 58 patients who were treated with lenvatinib between April 2018 and May 2021, had properly preserved serum at baseline, a best response point (in patients with SD, PR, CR) and PD point, and were evaluated for treatment response. Included patients were analyzed for serum growth factors at baseline and best response and PD points. We identified a total of 8, 24, 18, and 28 patients with CR, PR, SD, and PD, respectively (Figure 1).

The median age of the included patients was 70 years (range: 46–88 years), of which 52 (89.7%) patients were male. A total of 18, 11, and 29 patients had an etiology of hepatitis B virus infection, hepatitis C virus infection, and non-hepatitis B non-hepatitis C infection, respectively. A total of 43 (74.1%) patients had Child–Pugh grade A, and 35 (60.3%) had BCLC stage C. Of the 23 patients with BCLC B, 14 patients were refractory to transarterial chemoembolization (TACE), 7 patients were beyond the up-to-7 criteria, and 2 patients were TACE-unsuitable [24]. The median serum AFP level was 35 ng/mL (range: 1.6–449,909), and the median serum prothrombin induced by vitamin K absence-II level was 381 mAU/mL (range: 13–416,670). The overall median OS, PFS, and PPS were 148, 289, and 411 days, respectively (Figure S1).

### 3.2. Baseline Growth Factor Levels and Treatment Response

**Figure 2:**
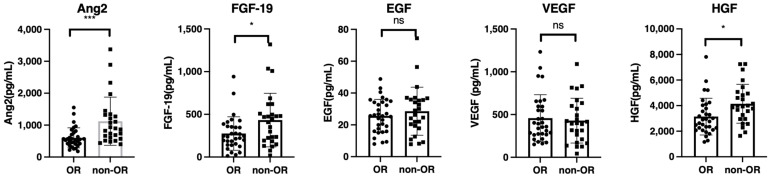
Comparison of baseline growth factors between patients with or without objective response (OR). Baseline serum FGF-19, ANG-2, HGF, EGF, and VEGF levels were compared between patients with or without an OR. Asterisks indicate statistically significant differences (* *p* < 0.05, *** *p* <0.001, ns: not significant). FGF-19, fibroblast growth factor-19; ANG-2, angiopoietin-2; HGF, hepatocyte growth factor; EGF, epidermal growth factor; VEGF, vascular endothelial growth factor.

As shown in Table 1, univariate analysis of baseline clinical factors revealed that age and RDI were significantly associated with objective response (OR). Subsequently, we analyzed the differences in baseline serum growth factors between patients with or without an OR. The growth factors levels were similar among the patients with HCC etiology of hepatitis B virus, hepatitis C virus, and non-B non-C hepatitis (Figure S2). As shown in Figure 2, baseline serum ANG-2, FGF-19, and HGF levels were significantly higher in patients without OR. Subsequently, we conducted a multiple logistic regression analysis for factors associated with OR. Age (odds ratio: 0.845; 95% confidence interval, 0.753–0.948; *p* = 0.004), RDI (odds ratio: 1.071; 95% confidence interval, 1.023–1.121; *p* = 0.003), and baseline ANG-2 levels (odds ratio: 0.996; 95% confidence interval, 0.993–0.999; *p* = 0.002) were significantly associated with OR in patients with unresectable HCC who were treated with lenvatinib (Table 2). In addition, the univariate and multivariate cox regression analysis revealed that baseline ANG2 and RDI were significantly associated with the PFS (Table S2)

HBV, hepatitis B virus infection; HCV, hepatitis C virus infection; NBNC, non-hepatitis B non-hepatitis C infection; BCLC, Barcelona Clinic Liver Cancer; AST, aspartate aminotransferase; ALT, alanine aminotransferase; Cr, creatinine; AFP, α-fetoprotein; PIVKA-II; protein induced by vitamin K absence-II; OR, objective response; RDI, relative dose intensity; MTA, molecular-targeted agents; TACE, transarterial chemoembolization; AE, adverse event; CH, chronic hepatitis; LC, liver cirrhosis; PD, progressive disease; RFA, radiofrequency ablation.

Subsequently, we analyzed baseline patient characteristics associated with disease control (SD, PR, and CR). As shown in Table S1, baseline platelet counts and AFP levels were significantly associated with a lack of disease control. However, significant differences were not observed in baseline growth factors between patients with or without disease controls (Figure S3).

Subsequently, we analyzed the changes in growth factors between baseline and PD points (*n* = 58). As shown in Figure S4, FGF-19, VEGF, and HGF levels significantly increased at the progressive point compared to those at baseline. Serum ANG-2 levels significantly decreased at the PD point. In addition, we analyzed the changes in the growth factors between baseline and best response point (*n* = 58). As shown in Figure S5, the FGF-19 and VEGF levels significantly increased at the best response point compared to those at baseline, while the serum ANG-2 levels significantly decreased at the best response point.

### 3.3. Changes in Growth Factors between Best Response and PD Points

**Figure 3:**
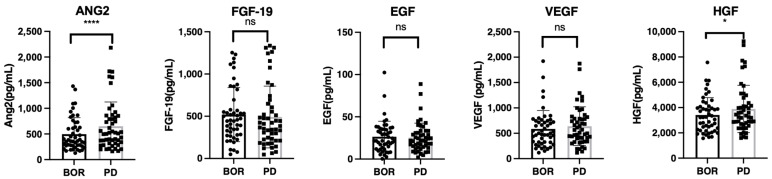
The changes in growth factors between the best response and progressive disease points in patients with SD, PR, and CR. Serum median FGF-19, ANG-2, HGF, EGF, and VEGF levels were compared between the best response and progressive disease points in patients with SD, PR, and CR. Asterisks indicate statistically significant differences (* *p* < 0.05, **** *p* < 0.0001, ns: not significant). BOR, best response; SD, stable disease; PR, partial response; CR, complete response.

To understand the changes in growth factors during HCC relapse after once responding to lenvatinib treatment, we analyzed the changes in growth factors between the best response and PD points (*n* = 49, SD = 18, PR = 24, CR = 7; one patient whose data of growth factors at the best response point could not be obtained was excluded). As shown in Figure 3, ANG-2 and HGF levels significantly increased at the PD point compared to those at the best response point.

### 3.4. Classification of Patients According to Clustering Analysis Based on Changes in Growth Factors

Recently, it was reported that the EGF/EGFR–PAK-2–ERK-5-mediated signaling pathway is involved in resistance to lenvatinib in patients with unresectable HCC [19]. Thus, we conducted clustering analysis based on the rate of changes in HGF and ANG-2, which significantly increased between the best response and PD points, and EGF.

**Figure 4:**
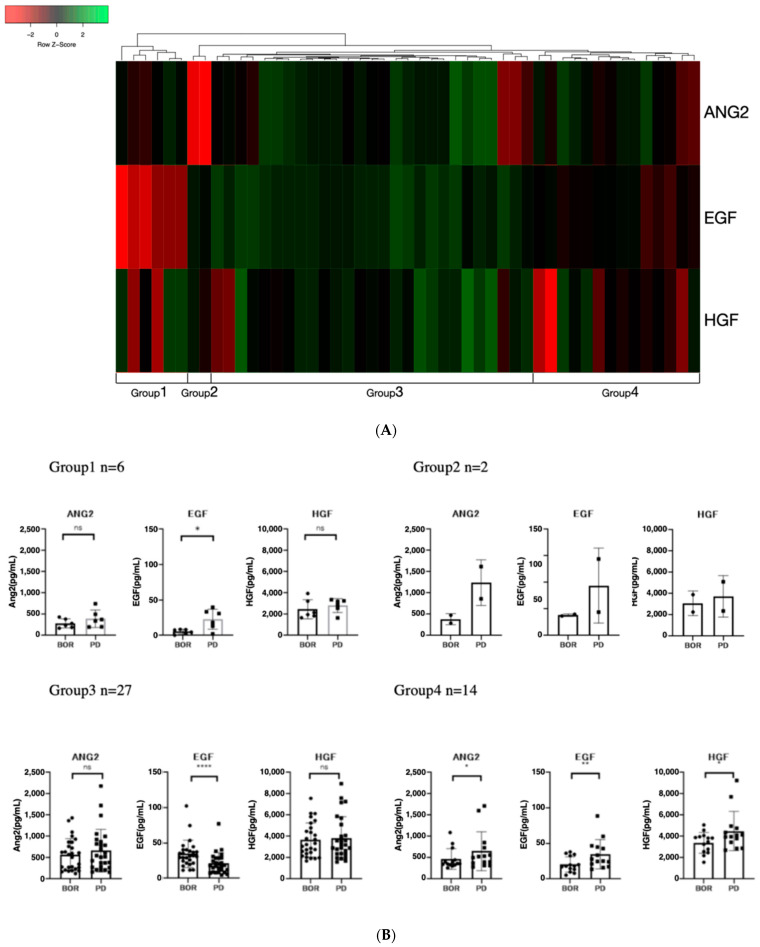

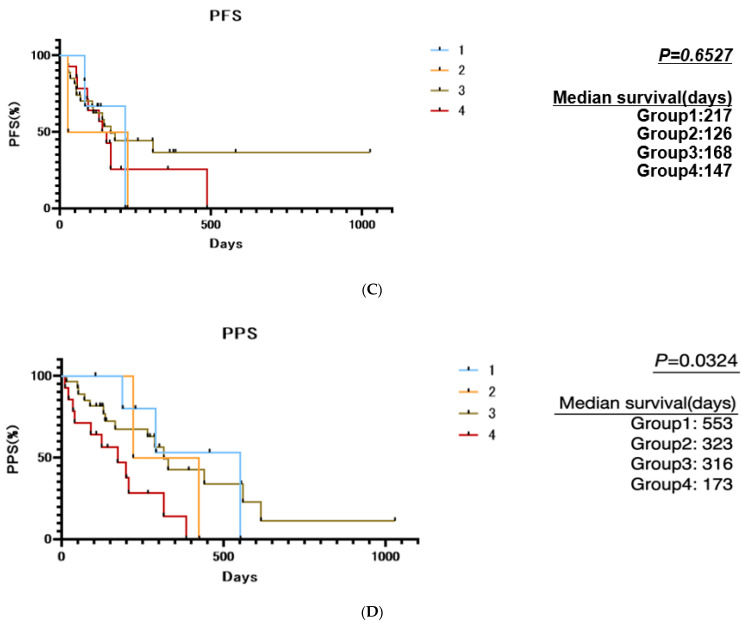
Cluster analysis based on the rate of changes in ANG-2, HGF, and EGF between best response and progressive disease points: (**A**) The rate of changes in EGF, ANG-2, and HGF between best response and progressive disease points in patients with SD, PR, and CR were visualized using a heat map and clustered by average linkage clustering methods using Heatmapper [23]. (**B**) Changes in ANG-2, HGF, and EGF between best response and progressive disease points in the four classified groups. Group 1 had a remarkable increase in EGF, group 2 had a remarkable increase in ANG-2, group 3 had no increase in growth factors but had a decrease in EGF, and group 4 had an increase in all three growth factors (ANG-2, HGF, and EGF). (**C**) Comparison of progression-free survival among the four classified groups based on rate of changes in ANG-2, HGF, and EGF between best response and progressive disease points. (**D**) Comparison of post progression survival among the four classified groups based on rate of changes in ANG-2, HGF, and EGF between best response and progressive disease points. Asterisks indicate statistically significant differences (* *p* < 0.05, ** *p* < 0.01, **** *p* < 0.0001, ns: not significant).

As shown in Figure 4A, based on clustering analysis, we classified the patients into four groups. As shown in Figure 4A,B, patients in group 1 (*n* = 6) had remarkably increased EGF levels between the best treatment response and PD points, those in group 2 (*n* = 2) had remarkably increased ANG-2 levels (*n* = 2), those in group 3 (*n* = 27) did not have increased ANG-2 and HGF levels but a substantial decrease in EGF level, and those in group 4 had significantly increased ANG-2, HGF, and EGF levels.

As shown in Table 3, Tables S3 and S4, and Figure 4C, the patient characteristics, including liver functional reserve (Child–Pugh class and ALBI score) and BCLC stage at baseline and at PD, the treatment response to lenvatinib, and the PFS were similar among the four groups. The best response and baseline growth factors were nearly similar among the four groups, except for the EGF levels at the best response point. The ANG2 levels, which are associated with OR, were similar among the four groups.

Importantly, as shown in Figure 4D, the PPS of the four groups was significantly different, and patients in group 4 had a much shorter median PPS than the other groups (median PPS in group 1 was 553 days, 323 days in group 2, 316 days in group 3, and 173 days in group 4; *p* = 0.0324).

## 4. Discussion

In this study, we analyzed sequential changes in growth factors at baseline and best response and PD points in lenvatinib treatment for patients with unresectable HCC. Multivariate analysis revealed that baseline ANG-2 levels, RDI, and age were significantly associated with the OR. Analysis of the changes in growth factors between the best treatment response and PD points revealed that, according to the rate of changes in EGF, ANG-2, and HGF levels, patients could be classified into four groups: one group with increased EGF, one group with increased ANG2, one group with no growth factor increase, and the last group with all growth factors increased. Although patient characteristics at baseline and PD point, treatment response to lenvatinib and PFS was similar among the four groups, and the patient group with all growth factors increased had a remarkably shorter PPS than the other groups (median PPS was 553 days, 323 days, 316 days, and 173 days for groups 1–4, respectively). These findings indicate that the pattern of resistance development to lenvatinib varies and might determine the prognosis of patients with unresectable HCC. Thus, the evaluation of baseline patient characteristics and the changes in growth factors during lenvatinib treatment could predict treatment response and PPS, and this information can be used to determine subsequent treatments.

It has been reported that OS is highly associated with PPS, not PFS, in patients with unresectable HCC undergoing sorafenib treatment [21]. Thus, a longer PPS is thought to be ideal for patients with unresectable HCC who are treated with TKIs. In this study, we classified patients who responded to lenvatinib based on the rate of changes in EGF, ANG-2, and HGF between the best treatment response and PD points. As shown in Figure 4, patients with increased median EGF, ANG-2, and HGF levels between the best treatment response and PD points had remarkably shorter PPS than other patient groups. A recent study revealed that the liver functional reserve is an important factor for PPS because sequential therapy after PD is an important factor for prolonging PPS [21]. Liver functional reserve was found to be similar among the four groups (Table 3 and Table S3). In addition, it has been reported that OR is associated with OS in systemic therapy for patients with unrespectable HCC [25]. In this study, the treatment responses among the four groups were similar. However, the cause of the remarkably shorter PPS in the patient group with increased median EGF, ANG2, and HGF than the other groups has not been clarified. Several hypotheses explaining this phenomenon have been proposed. High ANG-2 expression has been reported to be associated with rapid tumor growth, metastasis, and poor prognosis in HCC [26,27]. In addition, ANG-2 is associated with the progression of liver disease and fibrosis [28,29,30]. EGF plays a crucial role in the development of inflammation and causes the development of high metastatic potential in HCC [31]. Overexpression of HGF in HCC is associated with tumor invasion, metastasis, and the promotion of epithelial–mesenchymal transition [32]. Thus, simultaneous elevation of these three growth factors (ANG-2, HGF, and EGF) might cause an increase in tumors’ malignant potential, deterioration of liver function reserve, and resistance to salvage treatment. Further analyses are required to confirm the cause of the short PPS in group 4.

Several TKIs or anti-cancer drugs, including cabozantinib [4] and gefitinib [19], can inhibit HGF/c-MET-, ANG-2/Tie-2-, and/or EGF/EGFR-mediated signaling. These drugs might be effective for patients with unresectable HCC with increased EGF, ANG-2, and HGF levels between the best treatment response and PD points. Moreover, salvage therapy with lenvatinib plus gefitinib for some patients with unresectable HCC who are unresponsive to lenvatinib has been shown to elicit meaningful clinical responses [19]. Thus far, appropriate treatment for patients with unresectable HCC who fail to respond to lenvatinib has been an unmet clinical need. Thus, in addition to ICI therapy [33], TKIs with anti-EGF/EGFR, HGF/c-MET, and/or ANG-2/Tie-2 activity may be a potential therapeutic option. Further analysis is required to confirm this hypothesis.

Of baseline growth factors, multivariate analysis revealed that high ANG-2 levels were significantly associated with non-OR. Although lenvatinib could inhibit multi-kinase activity, including FGFR-1–4 and VEGFR, it could not inhibit ANG-2/Tie-2-mediated signaling. This result is consistent with previous studies showing that baseline ANG-2 levels are significantly associated with a poor response to lenvatinib in patients with unresectable HCC [11] and a shorter PFS in patients with advanced medullary thyroid cancer [17]. We previously reported that baseline ANG-2 levels do not affect the response to sorafenib treatment in patients with unresectable HCC [11]. Thus, in patients with unresectable HCC, the evaluation of baseline ANG-2 levels might be useful for the determination of therapeutic options. In this study, although we did not analyze the early changes in the growth factors, Chum et al. recently reported that the early changes in ANG-2 and FGF-19 could predict the response in lenvatinib for HCC [34]. Thus, further analysis, including early changes in each of the growth factors for lenvatinib treatment, is warranted in future studies.

This is the first report focusing on the sequential changes in growth factors during lenvatinib treatment in patients with unresectable HCC, especially changes between the best response and PD points. In addition, we revealed that these growth factors change during the development of resistance to lenvatinib, which affects PPS.

However, this study had a few limitations. It was a single-center retrospective study, and the number of patients was relatively limited. In addition, the included patients’ median age was relatively high, and the prevalence of male sex was high. Therefore, further validation in future prospective studies with larger sample sizes is required. Additionally, whether our findings are applicable to other systemic therapies for HCC remains to be investigated.

## 5. Conclusions

Our findings demonstrated that the pattern of development of resistance to lenvatinib varies and might determine the prognosis of patients with unresectable HCC. Thus, the evaluation of baseline patient characteristics and the changes in growth factors during lenvatinib could predict treatment response and PPS and can be used to determine subsequent treatments.

## Figures and Tables

**Table 1:** Baseline patient characteristics.

	Included Patients *n* = 58	OR (+) *n* = 32	OR (−) *n* = 26	*p*-Value
Age (years), median (range)	70 (46–88)	67 (47–83)	72 (46–88)	0.0159
Sex (male/female)	52/6	29/3	23/3	>0.9999
Etiology, *n* (%)				0.2130
HBV	18 (31.0%)	13 (40.6%)	5 (19.2%)	
HCV	11 (19.0%)	5 (15.6%)	6 (23.1%)	
NBNC	29 (50.0%)	14 (43.8%)	15 (57.7%)	
NBNC Etiology				0.3536
NAFLD	9 (31.0%)	5 (35.7%)	4 (26.7%)	
Alcohol	16 (55.2)	6 (42.9%)	10 (66.7%)	
Cryptogenic	4 (13.8%)	3 (21.4)	1 (6.7%)	
BCLC stage, *n* (%)				0.1063
B	23 (39.7%)	16 (50%)	7 (26.9%)	
C	35 (60.3%)	16 (50%)	19 (73.1%)	
Child–Pugh class, *n* (%)				0.2310
A	43 (74.1%)	26 (81.3%)	17 (65.4%)	
B	15 (25.9%)	6 (18.8%)	9 (34.6%)	
Child–Pugh score, *n* (%)				0.3894
5	23 (39.7%)	14 (43.8%)	9 (34.6%)	
6	20 (34.5%)	12 (37.5%)	8 (30.8%)	
≥7	15 (25.9%)	6 (18.8%)	9 (34.6%)	
Biochemical analysis				
Platelets, ×10^4^/μL	16.1 (4.4–138.0)	16.7 (6.5–50.0)	16.0 (4.4–138.0)	0.2862
AST, IU/L	37.5 (15–303)	35.0 (15–181)	51.0 (18–303)	0.0529
ALT, IU/L	25 (8–168)	25.0 (8–96)	25.0 (10–168)	0.6070
Cr, mg/dL	0.8 (0.3–1.7)	0.8 (0.5–1.4)	0.7 (0.3–1.7)	0.4818
AFP, ng/mL	35 (1.6–449,909.0)	10.4 (1.6–94,134.4)	206.8 (2.2–449,909.0)	0.0901
PIVKA-II, mAU/mL	381 (13–416,670.0)	373.0 (13.0–93,644.0)	389.0 (15.0–416,670.0)	0.1358
RDI	80.3 (6.3–100.0)	92.9 (50.0–100.0)	66.7 (6.3–100.0)	0.0003
History of MTA				0.7544
experienced	13 (22.4%)	8 (25.0%)	5 (19.2%)	
naïve	45 (77.6%)	24 (75.0%)	21 (80.8%)	
Cause of discontinuation				0.5105
AE	3 (23.1%)	1 (12.5%)	2 (40.0%)	
PD	10 (76.9%)	7 (87.5%)	3 (60.0%)	
Liver condition				0.3668
LC	53 (91.4%)	28 (87.5%)	25 (96.2%)	
CH	5 (8.6%)	4 (12.5%)	1 (3.8%)	
History of operation	20 (34.5%)	13 (40.6%)	7 (26.9%)	0.4053
History of RFA	14 (24.1%)	6 (18.8%)	8 (30.8%)	0.3611
History of TACE	33 (56.9%)	17 (53.1%)	16 (61.5%)	0.5991
History of systemic therapy	13 (22.4%)	8 (25.0%)	5 (19.2%)	0.7544
No treatment history	11 (19.0%)	6 (18.8)	5 (19.2%)	>0.9999

**Table 2:** Multivariate analysis for the factors associated with objective response (OR).

Multivariate Statistics Median (Range)	OR (+)	OR (−)	Multivariate Analysis	Odds Ratio 95% CI
ANG-2	542.7 (178.7–1550.9)	881.2 (401.0–3375.8)	*p* = 0.002	0.996 (0.993–0.999)
HGF	2832.9 (1166.1–7811.4)	3963.7 (1635.5–7257.9)	ns	-
FGF-19	241.5 (13.0–941.8)	428.2 (19.1–1319.0)	ns	-
Age	67 (47–83)	72 (46–88)	*p* = 0.004	0.845 (0.753–0.948)
RDI	92.9 (50.0–100.0)	66.7 (6.3–100.0)	*p* = 0.003	1.071 (1.023–1.121)

ANG-2, angiopoietin-2; HGF, hepatocyte growth factor; FGF-19, fibroblast growth factor-19. RDI, relative dose intensity.

**Table 3:** Comparison of patient characteristics at progressive disease point among the four groups classified according to changes in growth factors between best response and progressive disease points.

	Group 1; *n* = 6	Group 2; *n* = 2	Group 3; *n* = 27	Group 4; *n* = 14	*p*-Value
Age (years), median (range)	70 (54–80)	74.5 (66–83)	68 (47–88)	70.5 (54–83)	0.7518
BCLC stage, *n* (%)					0.3494
B	2 (33.3%)	0 (0.0%)	12 (44.4%)	3 (21.4%)	
C	4 (66.7%)	2 (100.0%)	15 (55.6%)	11 (78.6%)	
Child–Pugh class, *n* (%)					0.8629
A	5 (83.3%)	2 (100.0%)	18 (66.7%)	8 (57.1%)	
B	1 (16.7%)	0 (0.0%)	8 (29.6%)	5 (35.7%)	
C	0 (0.0%)	0 (0.0%)	1 (3.7%)	1 (7.1%)	
Child–Pugh score, *n* (%)					0.5814
5	4 (66.7%)	1 (50.0%)	8 (29.6%)	4 (28.6%)	
6	1 (16.7%)	1 (50.0%)	10 (37.0%)	4 (28.6%)	
≥7	1 (16.7%)	0 (0.0%)	9 (33.3%)	6 (42.9%)	
Biochemical analysis, median (range)					
Platelets, ×10^4^/μL	14.0 (3.1–13.6)	8.6 (7.1–10.1)	14.0 (4.4–56.6)	15.5 (9.1–37.6)	0.4856
AST, IU/L	40.5 (31–136)	56 (54–58)	41 (16–278)	58.5 (23–568)	0.5051
ALT, IU/L	26.0 (17–172)	29 (10–48)	24 (8–56)	42.5 (19–209)	0.0728
Cr, mg/dL	1.0 (0.7–1.2)	0.8 (0.5–1.0)	0.9 (0.4–2.0)	0.9 (0.5–2.4)	0.8979
ALB	3.6 (3.2–4.1)	3.3 (2.9–3.6)	3.4 (2.3–4.2)	3.2 (2.2–7.1)	0.8456
T-Bil	1.1 (0.5–3.5)	1.5 (1.4–1.6)	0.9 (0.4–3.8)	0.85 (0.4–10.7)	0.7171
ALBI score	−2.2 (−2.9–(−1.5))	−1.83 (−2.1–(−1.5))	−2.1 (−3.0–(−1.0))	−1.7 (−5.5–(−0.6))	0.9212
AFP, ng/mL	179.8 (1.9–5,288.8)	63 (4.6–121.4)	31.65 (1.7–191,182)	12.95 (2.6–95,909.8)	0.8305
PIVKA-II, mAU/mL	386.5 (22.0–10,744.0)	20,513.5 (474.0–40,553.0)	2930 (21–548,521)	2853 (15–57,558)	0.8827
Treatment response of lenvatinib					0.6711
CR	0 (0.0%)	0 (0.0%)	4 (14.8%)	3 (21.4%)	
PR	4 (66.7%)	1 (50.0%)	11 (40.7%)	8 (57.1%)	
SD	2 (33.3%)	1 (50.0%)	12 (44.4%)	3 (21.4%)	

ALB, albumin; T-Bil, total bilirubin; ALBI, albumin–bilirubin; BCLC, Barcelona Clinic Liver Cancer; CR, complete response; PR, partial response; SD, stable disease.

## Data Availability

The data that support the findings of this study are available from the corresponding author upon reasonable request.

## Supplementary Materials

The following supporting information can be downloaded at https://www.mdpi.com/article/10.3390/cancers14010232/s1, Figure S1: Progression-free survival, post progression survival, and overall survival among all the patients, Figure S2: Comparison of the growth factor levels among patients with hepatocellular carcinoma (HCC) etiology of hepatitis B virus, hepatitis C virus, and non-B non-C hepatitis, Figure S3: Comparison of baseline growth factors between patients with and without disease control (CR, PR, SD), Figure S4: The changes in growth factors between baseline and progressive disease points, Figure S5: The changes in the growth factors between baseline and best response points, Table S1: Comparison of baseline patient characteristics between patients with and without disease control (DC), Table S2: The factors associated with progression-free survival, Table S3: Comparison of patient characteristics at baseline among the four groups classified according to changes in growth factors between best response and progressive disease points, Table S4: Comparison of the growth factor levels of the four groups classified according to the changes in the growth factors at baseline and best response.
