# Supplementary material for: Changes in Serum Growth Factors during Lenvatinib Predict the Post Progressive Survival in Patients with Unresectable Hepatocellular Carcinoma
Source: Cancers (Basel). 2022 Jan 4;14(1):232. doi: 10.3390/cancers14010232 (PMC8750627; doi:10.3390/cancers14010232)
Supplement: Supplementary file 1 [file cancers-14-00232-s001.zip › supplementary-Figures.pdf]

cancers

MDPI

Supplementary figures

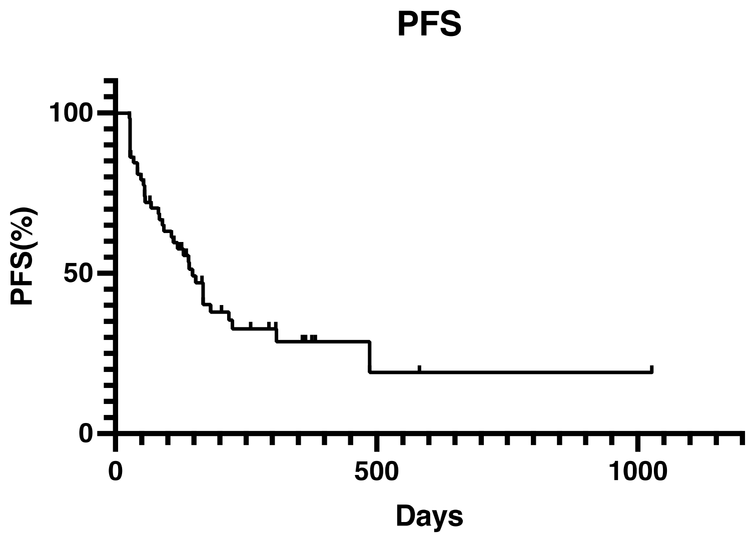

A

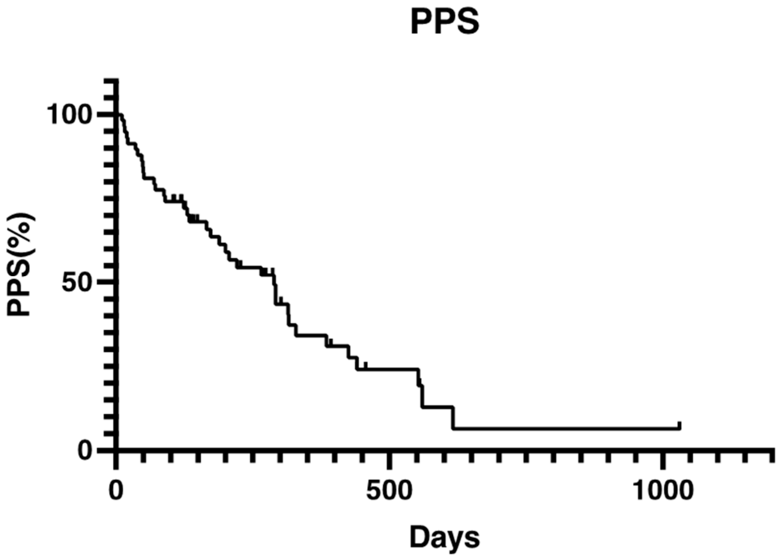

B

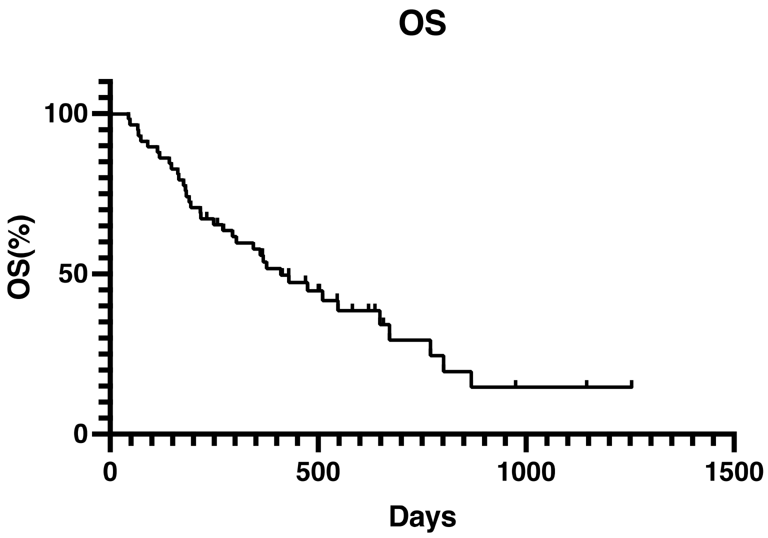

C

Figure S1: Progression-free survival, post-progression survival, and overall survival among all the patients **A.** Progression-free survival (n=58) **B**. Post-progression survival (n=58) **C**. Overall survival(n=58)

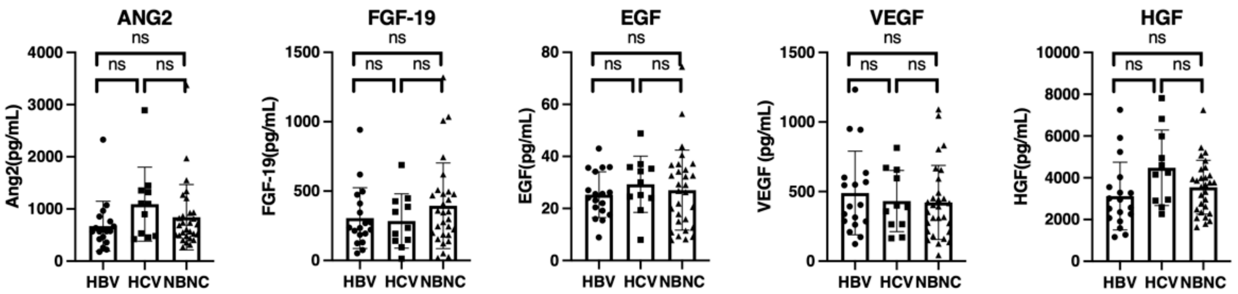

P=0.1983 P=0.3790 P=0.7174 P=0.6919 P=0.0687

Figure S2: Comparison of the growth factor levels among the patients with hepatocellular carcinoma (HCC) etiology of hepatitis B virus, hepatitis C virus, and non-B non-C hepatitis. Serum median FGF-19, ANG-2, HGF, EGF, and VEGF levels were compared among the patients with HCC etiology of hepatitis B virus, hepatitis C virus, and non-B non-C hepatitis. FGF-19, fibroblast growth factor-19; ANG-2, angiopoietin-2; HGF, hepatocyte growth factor; EGF, epidermal growth factor; VEGF, vascular endothelial growth factor.

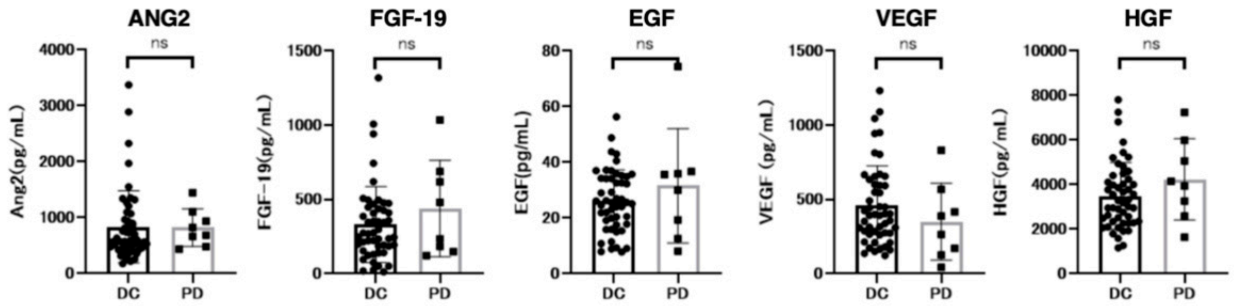

Figure S3: Comparison of baseline growth factors between patients with or without disease control (CR, PR, SD). Baseline serum FGF-19, ANG-2, HGF, EGF, and VEGF levels were compared between patients with or without disease control (CR, PR, and SD). Asterisks indicate statistically significant differences (* $p < 0.05$, ** $p < 0.01$, *** $p < 0.001$, **** $p < 0.0001$). FGF-19, fibroblast growth factor-19; ANG-2, angiopoietin-2; HGF, hepatocyte growth factor; EGF, epidermal growth factor; VEGF, vascular endothelial growth factor; DC, disease control; PD, progressive disease

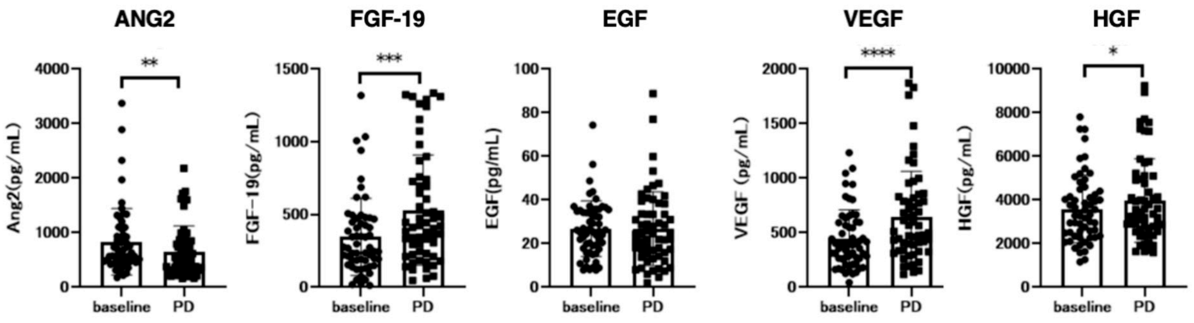

Figure S4: The changes in growth factors between baseline and progressive disease points. Serum median FGF-19, ANG-2, HGF, EGF, and VEGF levels were compared between baseline and progressive disease points in all cohorts. Asterisks indicate statistically significant differences (* $p < 0.05$,** $p < 0.01$, *** $p < 0.001$, **** $p < 0.0001$). FGF-19, fibroblast growth factor-19; ANG-2, angiopoietin-2; HGF, hepatocyte growth factor; EGF, epidermal growth factor; VEGF, vascular endothelial growth factor; DC, disease control; PD, progressive disease

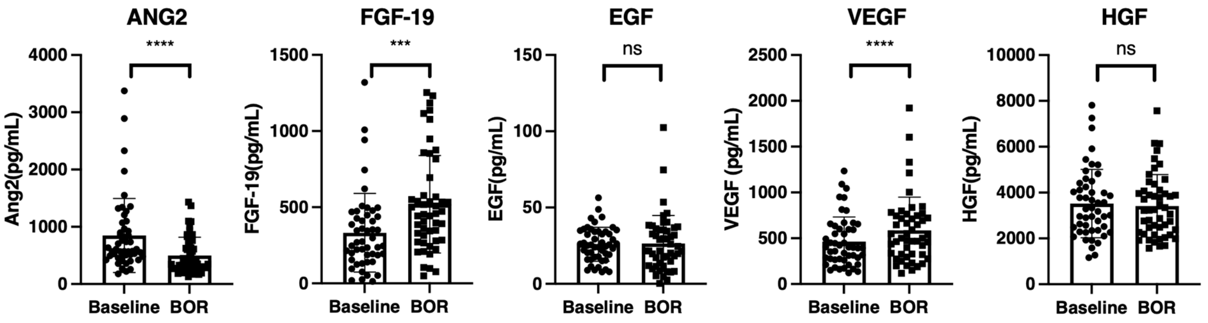

Figure S5: The changes in the growth factors between the baseline and best response points. Serum median FGF-19, ANG-2, HGF, EGF, and VEGF levels were compared between the baseline and best response points in all the cohorts. Asterisks indicate statistically significant differences (* $p < 0.05$,** $p < 0.01$, *** $p < 0.001$, **** $p < 0.0001$). FGF-19, fibroblast growth factor-19; ANG-2, angiopoietin-2; HGF, hepatocyte growth factor; EGF, epidermal growth factor; VEGF, vascular endothelial growth factor; BOR, best response.
