# Supplementary material for: Changes in Serum Growth Factors during Lenvatinib Predict the Post Progressive Survival in Patients with Unresectable Hepatocellular Carcinoma
Source: Cancers (Basel). 2022 Jan 4;14(1):232. doi: 10.3390/cancers14010232 (PMC8750627; doi:10.3390/cancers14010232)
Supplement: Supplementary file 1 [file cancers-14-00232-s001.zip › Supplementary-Tables.pdf]

cancers

MDPI

Supplementary Tables

Table S1. Comparison of baseline patient characteristics between patients with and without disease control (DC).

	DC(+) n=50	DC(-) n=8	P-value
**Age (years), median (range)**	70 [47-88]	74 [46-82]	0.4245
**Sex (male/female)**	46/4	6/2	0.1886
**Etiology, n (%)**			0.2582
**HBV**	17 [34.0%]	1 [12.5%]	
**HCV**	8 [16.0%]	3 [37.5%]	
**NBNC**	25 [50.0 %]	4 [50.0%]	
**BCLC stage, n (%)**			0.1290
**B**	22 [44.0%]	1 [12.5%]	
**C**	28 [56.0%]	7 [87.5%]	
**Child–Pugh class, n (%)**			0.4139
**A**	38 [76.0%]	5 [62.5%]	
**B**	12 [24.0%]	3 [37.5%]	
**Child–Pugh score, n (%)**			0.6911
**5**	20 [40.0%]	3 [37.5%]	
**6**	18 [36.0%]	2 [25.0%]	
**≥7**	12 [24.0%]	3 [37.5%]	
**Biochemical analysis, median (range)**			
**Platelets**	16.1 [4.4-51.7]	18.0 [7.3-138.0]	0.0115
**AST, IU/L**	37.0 [15.0-303.0]	51.0 [22.0-75.0]	0.6392
**ALT, IU/L**	25.0 [8.0-168.0]	24.5 [10.0-65.0]	0.5923
**Cr, mg/dL**	0.8 [0.3-1.7]	0.7 [0.6-1.1]	0.3543
**AFP, ng/mL**	26.5 [1.6-254560.8]	860.8 [4.2-449909.0]	0.0026
**PIVKA-II, mAU/mL**	413.0 [13.0-416670.0]	140.0 [15.0-27425.0]	0.5722

HBV, hepatitis B virus infection; HCV, hepatitis C virus infection; NCBC, non-hepatitis B non-hepatitis C infection; BCLC, Barcelona Clinic Liver Cancer; AST, aspartate aminotransferase; ALT, alanine aminotransferase; Cr, creatinine; AFP, α-fetoprotein; PIVKA-II; protein induced by vitamin K absence-II.

Table S2. The factors associated with progressive free survival.

	Univariate analysis		Multiple analysis	
	HR (95% CI)	*P-value*	HR (95% CI)	*P-value*
**Age, years**	1.01	0.5313		
**Sex**	0.55	0.1737		
**HBV positive**	0.8967	0.7102		
**HCV positive**	1.385	0.3412		
**NBNC**	0.9117	0.9117		
**BCLC B**	0.8877	0.6643		
**BCLC C**	1.126	0.6643		
**Child-Pugh class A**	0.6993	0.2408		
**Child-Pugh class B**	1.43	0.2408		
**Child-Pugh score 5**	0.7119	0.217		
**Child-Pugh score 6**	0.217	0.7514		
**Child-Pugh score ≥7**	1.43	0.2408		
**Platelets, ×10⁴/μL**	0.9995	0.9387		
**AST, IU/L**	1.003	0.2031		
**ALT, IU/L**	1.002	0.7114		
**Cr, mg/dL**	0.5513	0.2766		
**AFP, ng/mL**	1	0.207		
**PIVKA-II, mAU/mL**	1	0.3603		
**RDI**	0.989	0.03382	1.0010	0.01788

**Ang-2, pg/mL**	1	0.0312	0.9881	0.02028
**FGF-19, pg/mL**	1	0.67		
**EGF, pg/mL**	1.024	0.06666		
**VEGF, pg/mL**	0.9995	0.3446		
**HGF, pg/mL**	1	0.1369		

HBV, hepatitis B virus infection; HCV, hepatitis C virus infection; NCBC, non-hepatitis B non-hepatitis C infection; BCLC, Barcelona Clinic Liver Cancer; AST, aspartate aminotransferase; ALT, alanine aminotransferase; Cr, creatinine; AFP, α-fetoprotein; PIVKA-II; protein induced by vitamin K absence-II.

Table S3. Comparison of patient characteristics at baseline among the four groups classified according to changes in growth factors between best response and progressive disease points.

	Group 1 n=6	Group 2 n=2	Group 3 n=27	Group 4 n=14	P-value
**Age (years), median (range)**	70(55-81)	74.5(66-83)	67(47-88)	70(54-83)	0.7174
**Sex (male/female)**	4/2	2/0	25/2	14/0	0.0891
**Etiology, n (%)**					0.5711
HBV	3(50.0%)	0(0.0%)	8(29.6%)	6(42.9%)	
HCV	0(0.0%)	0(0.0%)	5(18.5%)	3(21.4%)	
NBNC	3(50.0%)	2(100.0%)	14(51.9%)	5(35.7%)	
**BCLC stage, n (%)**					0.5660
B	2(33.3%)	0(0.0%)	13(48.1%)	6(42.9%)	
C	4(66.7%)	2(100.0%)	14(51.9%)	8(57.1%)	
**Child-Pugh class, n (%)**					0.7762
A	4(66.7%)	2(100.0%)	21(77.8%)	10(71.4%)	
B	2(33.3%)	0(0.0%)	6(22.2%)	4(28.6%)	
**Child-Pugh score, n (%)**					0.8802
5	3(50.0%)	1(50.0%)	11(40.7%)	4(28.6%)	
6	1(16.7%)	1(50.0%)	10(37.0%)	6(42.9%)	
≥7	2(33.3%)	0(0.0%)	6(22.2%)	4(28.6%)	
			**Biochemical analysis, median(range)**		
Platelet	16.7(7.8-22.1)	13.8(8.5-19.1)	16.0(4.4-51.7)	16.1(6.5-50.0)	0.7431
AST, IU/L	37.0(25.0-90.0)	51.0(44.0-58.0)	34.0(18.0-303.0)	40.0(23.0-179.0)	0.9372
ALT, IU/L	23.5(21.0-39.0)	28.5(15.0-42.0)	24.0(13.0-144.0)	31.0(13.0-168.0)	0.6142
Cr, mg/dL	0.8(0.5-0.9)	0.7(0.6-0.9)	0.8(0.3-1.7)	0.8(0.5-1.4)	0.5877
AFP, ng/mL	102.3(2.0-17832.0)	51.8(6.1-97.5)	8.9(1.6-254560.8)	28.0(2.2-94134.4)	0.8977
PIVKA-II, mAU/mL	66.5(24.0-23996.0)	2947.0(373.0-5521.0)	654.0(18.0-416670.0)	422.0(13.0-195319.0)	0.9439

HBV, hepatitis B virus infection; HCV, hepatitis C virus infection; NCBC, non-hepatitis B non-hepatitis C infection; BCLC, Barcelona Clinic Liver Cancer; AST, aspartate aminotransferase; ALT, alanine aminotransferase; Cr, creatinine; AFP, α-fetoprotein; PIVKA-II; protein induced by vitamin K absence-II.

Table S4. Comparison of growth factors levels of four groups classified according to changes in growth factors at baseline and best response.

	**Group 1; n=6**	**Group 2; n=2**	**Group 3; n=27**	**Group 4; n=14**	**P-value**
			**Baseline Growth factors level, average (range)**		
**ANG-2 (pg/mL)**	469..2 [266.9-634.0]	708.1 [552.2-864.0]	922.3 [178.7-3375.8]	888.9 [357.0-2890.7]	0.4764
**FGF-19(pg/mL)**	257.8 [154.9-475.0]	182.1 [139.4-224.8]	393.4 [19.1-1319.0]	267.8 [13.0-410.6]	0.3183
**EGF (pg/mL)**	25.2 [15.0-40.6]	27.9 [26.8-29.1]	26.6 [7.9-56.4]	24.7 [8.9-48.8]	0.9526
**VEGF (pg/mL)**	265.5 [122.9-449.6]	234.2 [151.1-317.3]	487.1 [136.5-1090.2]	532.9 [161.0-1233.4]	0.1176
**HGF (pg/mL)**	2685.8 [1166.1-3862.5]	3167.6 [2762.4-3572.7]	3816.1 [1586.9-7811.4]	3356.9 [1268.8-5909.2]	0.3693
			**BOR Growth factors level, average (range)**		
**ANG-2 (pg/mL)**	282.4 [169.2-432.7]	383.2 [290.5-475.9]	569.7 [127.7-1431.3]	467.1 [260.7-1091.1]	0.2195
**FGF-19 (pg/mL)**	447.3 [224.4-1077.2]	904.6 [576.8-1232.4]	550.0 [77.5-1253.0]	437.3 [49.7-876.0]	0.2270
**EGF (pg/mL)**	5.3 [0.3-8.6]	21.5 [21.5-21.6]	34.6 [11.5-102.4]	20.5 [5.3-38.1]	0.0008
**VEGF (pg/mL)**	313.4 [116.8-552.0]	381.6 [436.1-327.2]	688.5 [199.0-1921.3]	533.3 [222.4-744.2]	0.0858
**HGF (pg/mL)**	2464.8 [1665.5-3943.4]	3081.0 [2264.1-3898.0]	3662.1 [1686.0-7574.3]	3398.8 [1568.0-5070.0]	0.2825

FGF-19, fibroblast growth factor-19; ANG-2, angiopoietin-2; HGF, hepatocyte growth factor; EGF, epidermal growth factor; VEGF, vascular endothelial growth factor; BOR; best response; PD, progressive disease.
